# Treatment of recurrent epileptic seizures in patients with neurological disorders

**DOI:** 10.3892/etm.2012.788

**Published:** 2012-11-01

**Authors:** GUAN-QIAN YUAN, DAN-DAN GAO, JUN LIN, SONG HAN, BO-CHUANG LV

**Affiliations:** Department of Neurosurgery, General Hospital of Shenyang Military Area Command of Chinese PLA, Shenyang, Liaoning 110016, P.R. China

**Keywords:** epileptic seizure, treatment, levetiracetam

## Abstract

This study aimed to investigate the clinical characteristics and the treatment principles and methods of recurrent epileptic seizures in patients with neurological disorders. A retrospective analysis was performed of the clinical data, treatment methods and results in 13 patients with recurrent epileptic seizures attending the neurosurgery department. Of the 13 patients, 10 had a history of epilepsy, 9 had organic frontal lobe brain lesions and 11 exhibited frontal lobe epilepsy. The causes of the epileptic seizure aggravation included drug withdrawal, dose reduction and dressing change (5 cases). The epileptic seizure types included partial and secondary full seizures and the seizure frequency ranged from 1 seizure/3 min to 1 seizure/several h. Following combined therapy with multiple anti-epileptic drugs (AEDs), including oral administration and injection, the epilepsy was controlled. The addition of orally administered levetiracetam improved the treatment efficiency. In cases of recurrent epileptic seizures in patients with neurological disorders, the combined administration of AEDs should be conducted with doses higher than the conventional initial dose to control the epileptic seizures as rapidly as possible.

## Introduction

Epilepsy is a common disease of the nervous system and domestic epidemiology surveys show that its incidence rate is 0.4–0.7%. Certain neurological disorders are frequently complicated with epilepsy, including cerebral tumors, arteriovenous malformation, cavernous hemangioma, craniocerebral injury and cerebral cortical dysplasia. The mechanism involves lesions which damage the brain structure and impair the nerve conduction function (1). Among supratentorial brain gliomas, 30–40% are complicated with epilepsy (1,2) and the epilepsy incidence rate of low-grade gliomas, such as oligodendroglioma, may be up to 70% (3,4).

At present, the treatment principle for anti-epileptic drugs (AEDs) is to select the correct AED according to the epileptic seizure type and epileptic syndrome classification (5,6). Monotherapy is preferred. If the first monotherapy fails, it is replaced by a second monotherapy. In cases where the second monotherapy does not control the seizures, combined therapy is used (7,8). To relieve or reduce the adverse reactions or side effects of AEDs, a small dose of AED is used initially and the dose is gradually increased once every 1 or 2 weeks. For certain AEDs, 4 to 8 weeks are required to reach the effective dose (9).

For patients with epilepsy, a variety of factors aggravate the epileptic seizures and result in status epilepticus (SE). SE is defined as unrecovered consciousness in the intermission between seizures for generalized-seizure patients with a loss of consciousness. It is a critical condition of the nervous system and hypoxia or other causes may result in encephaledema. Following neurosurgery, SE in patients may aggravate encephaledema and induce serious consequences. Therefore, it is necessary to conduct emergency treatment for SE to rapidly terminate the seizure. The most common drug for terminating seizures is diazepam which is administered at a dose of 10–20 mg via intravenous injection and an injection speed <2–5 mg/min (10,11).

For some patients with epilepsy complicated with neurological disorders, epileptic seizures may be aggravated. The epileptic seizures reoccur repeatedly and the frequency may be as high as several hundred in one day. The disease condition of these patients is more severe than general epileptic seizures but does not comply with the diagnosis criteria of SE. Moreover, treatment is extremely difficult. If the epileptic seizure cannot be controlled quickly, encephaledema, hemorrhage and other serious consequences may occur. At present, no studies concerning the diagnosis and treatment of this type of epileptic seizure have been reported. The present study describes the clinical characteristics and treatment methods of 13 patients with recurrent epileptic seizures treated between January 2011 and June 2012.

## Materials and methods

### General data

The present study included patients in the Department of Neurosurgery of the General Hospital of Shenyang Military Area Command of Chinese PLA who were admitted due to recurrent epileptic seizures or who exhibited recurrent epileptic seizures during treatment. The numbers of epileptic seizures reached >10 per day. There were 8 male and 5 female patients who were between 23 and 53 years old. The average age was 41.6 years old. Also, 10 patients had a previous history of epilepsy and the disease course was 2–36 years. Of the patients, 3 had no history of epilepsy, 1 had a history of head trauma, 2 had a history of right-frontal glioma resection, 1 had a 32-year history of left-frontal lobe arachnoid cyst surgery and 1 had a history of frontal meningioma resection.

During hospitalization, a conventional 10/20 system electroencephalogram examination was performed for 6 patients and video electroencephalogram monitoring (V-EEG) was conducted for 7. A head CT examination was performed for 5 patients, head MRI examination for 3 and both CT and MRI examinations were performed for the remaining 5. The present study was conducted in accordance with the Declaration of Helsinki and with approval from the Ethics Committee of the General Hospital of Shengyang Military Area Command of Chinese PLA. Written informed consent was obtained from all participants.

### Treatment

Ten of the patients were treated with multiple drugs due to recurrent epileptic seizures but the treatment did not effectively in control the seizures. Therefore, the patients were transferred to the Department of Neurosurgery from the hospital’s emergency department or from external hospitals. AEDs were regulated for all patients according to the epileptic seizure type and previous treatment with AEDs. In more severe cases, intramuscular or intravenous injections were performed. The specific treatments are shown in Table I. In cases of generalized tonic clonic seizure (GTCS) and non-convulsive SE, diazepam (10 mg) was administered via intravenous injection and the dose was gradually reduced according to the disease condition following seizure control.

## Results

### Imaging

Imaging results indicated that there were 2 cases of right-frontal lobe gliomas, 2 cases of right-frontal glioma recurrences, 1 left-frontal lobe arachnoid cyst, 1 right-frontal cavernous hemangioma, 1 left-frontal encephalomalacia, 1 bilateral-frontal cerebral softening lesion and 1 right-frontal cerebral infarction.

### Reasons for recurrent epileptic seizures

Four cases reoccurred due to the decrease or withdrawal of AEDs, 1 due to the change of AEDs, 2 due to newly diagnosed cerebral tumors, 1 due to surgery (intracranial electrode implantation), 1 due to cerebral infarction and 4 due to unknown causes.

### Types and frequencies of epileptic seizures

Among the patients, there were 3 cases of simple partial seizures, 4 of complex partial seizures, 2 of complex partial seizures plus secondary grand mal seizures, 1 of simple partial seizure plus complex partial seizure, 1 of simple partial seizure plus secondary grand mal seizure and 2 of complex partial seizures plus non-convulsive status. In addition, 11 cases exhibited frontal lobe epilepsy and 2 exhibited temporal lobe epilepsy. Epileptic seizure frequencies were from 1 seizure/3 min to 1 seizure/several hours and the maximum number of seizures within one day was in the hundreds.

### Types and doses of AEDs and treatment results

For all 13 patients, a variety of current common first- or second-line AEDs were jointly used for treatment. If seizure control was unsatisfactory and epilepsy reoccurred within 1–4 days after the administration, levetiracetam (LEV; 0.5, 1.0, or 2 g bid) was administered and an intravenous injection of sodium valproate and intramuscular injection of phenobarbital sodium were administered. As a result, epileptic seizures were controlled. Oral sodium valproate (500 mg, tid) oxcarbazepine (Qulai, 150 mg, bid), LEV (1.0, bid) and phenobarbital sodium (0.2 g, bid, intramuscular injection) were administered to 1 patient. One day later, epilepsy still frequently reoccurred so the dose of oxcarbazepine was increased to 300 mg, bid and the epileptic seizures were controlled the same day. Prior to hospitalization, one patient did not receive AEDs. Following monotherapy, valproic acid (VPA) was administered for 2 days, and the seizures were controlled. As a result of the treatment, the recurrent epileptic seizures were controlled and the time between seizures was 10 h and 5 days.

## Discussion

In the patients of the present study, the number of epileptic seizures significantly increased in a short time and reoccurred repeatedly. For the cases without a previous history of epilepsy, epilepsy occurred suddenly, a number of times. The patient with the maximum number of epileptic events experienced hundreds of seizures. The epileptic seizure types were mostly complex partial seizures and simple partial seizures, but a number were complicated with secondary GTCS. However, the convulsion duration was shorter and there was no apparent consciousness disorder, which differs from SE (10,11).

In the present study, 10 patients had a history of epilepsy (76.9%). Among the 13 patients, 9 had clear organic lesions located in the frontal lobe. Epileptic seizure symptoms and electroencephalogram examination revealed 11 cases of frontal lobe epilepsy and 2 of temporal lobe epilepsy, suggesting that the patients with complications due to organic pathological changes exhibited more aggravated epileptic seizures and frontal lobe lesions (2,12). Compared with the other lobes of brain, the frontal lobe is responsible for more epileptic and recurrent epileptic seizures.

With regard to the causes of the recurrent epileptic seizures in the present study, 5 cases reoccurred due to drug withdrawal, dose reduction or dressing change. These were the most common reasons. Cerebral tumors, tumor recurrence following surgery and surgery were also possible causes. The causes of recurrent epileptic seizures are similar to those of SE (drug withdrawal, dose reduction, dressing change, fever and infection) (10,11).

Recurrent epileptic seizures may cause acute damage to the nervous system and certain seizures become SE. Recurrent epileptic seizures are capable of aggravating encephaledema, delaying the recovery of consciousness and causing brain herniation due to the aggravated encephaledema, particularly in neurosurgery patients in the perioperative period. Therefore, it is necessary to control recurrent epileptic seizures as rapidly as possible.

In the present study, for the initial treatment of patients following the onset of recurrent epileptic seizures, combined therapy with various AEDs was conducted. Traditional and novel AEDs, as well as sodium valproate injections, were administered but not all of the seizures were effectively controlled. As the types and doses of AEDs were modified, the seizures were gradually controlled, although the treatment duration of certain patients was longer at 5 days. At present, there are no clear diagnostic criteria and treatment guidelines for recurrent epileptic seizures. However, recurrent epileptic seizures are a common clinical situation and it is necessary to control the seizures as rapidly as possible in order to reduce neurological complications and the mortality rate. As for the treatment principle, in cases of common epilepsy, monotherapy is adopted and the dose is increased from a small dose to the effective treatment dose. It is possible that the epileptic seizures may not be effectively controlled within several weeks to several months, which delays the patient’s condition. However, recurrent epileptic seizures differ from SE in that between the seizures, the patient is conscious. Also, the severity is milder than that of SE. For the treatment of SE, intravenous injections of diazepam are preferred. However, the action time of diazepam is short. Therefore, it is more suitable for simple epileptic grand mal seizures rather than for partial seizures. In the present study, after diazepam was injected, the simple epileptic seizures of the patients stopped, but subsequently, epileptic seizures reoccurred repeatedly in some cases. Consequently diazepam is not an ideal drug for recurrent epileptic seizures.

LEV (Kaipulan) is a relatively novel AED. Multiple placebo-controlled (13–15) and open-label (16–18) clinical studies suggest that, as an additional therapy, LEV has good treatment efficiency for refractory partial epilepsy of children and adults and high tolerance and safety. LEV also has ideal pharmacokinetic characteristics. The absorption of oral LEV is complete (>95%) and it has the advantages of high bioavailability (close to 100%), linear pharmacokinetics, low protein binding rate, low hepatic metabolism and reduced drug interactions. Moreover, it rapidly achieves a stable blood drug concentration. Absorption of oral LEV is rapid and the peak plasma concentration is achieved within 0.6 to 1.3 h. Moreover, LEV may be administered at a high dose in the initial treatment and there are few specific side effects (19). For the patients in the present study, the addition of LEV improved the treatment efficiency for recurrent epileptic seizures, which was possibly associated with its marked anti-epileptic effect and the rapid absorption and action following oral administration.

The clinical characteristics and treatment methods of recurrent epileptic seizures are different from those of common epilepsy and SE. For the treatment of recurrent epileptic seizures, in cases of focal epileptic seizures, combined AED therapy has a high treatment efficiency. The doses of the AEDs, including those for intravenous and intramuscular injection, should be higher than the conventional initial doses, for the early control of the epileptic seizures.

## Figures and Tables

**Table I:** Clinical data of 13 patients with recurrent epileptic seizures.

Case	Gender	Age (years)	Seizure types	Initial administration	Later administration (additional)
1	Male	38	Complex partial seizure	Carbamazepine, 0.2, tid; sodium valproate injection, 400 mg IV, q8 h; sodium valproate tablet, 500 mg, tid; sodium valproate injection, 400 mg IV, q12 h; phenobarbital sodium 0.2 IM, bid	Kaipulan 1.0, bid
2	Female	53	Complex partial seizure		Kaipulan 1.0, bid
3	Male	27	Complex partial seizure, non-convulsive status epilepticus	Qulai 600 mg bid; topiramate, 50 mg, bid; phenobarbital sodium, 0.2 IM bid; sodium valproate 400 mg IV, q8 h	Kaipulan 1.0, bid
4	Male	34	Simple partial seizure, Complex partial seizure	Sodium valproate 500 mg, tid; kaipulan 1.0, bid; Qulai 150 mg, bid; phenobarbital, 0.2 IM, bid	Change into Qulai, 300 mg, bid
5	Male	31	Complex partial seizure, non-convulsive status epilepticus	Sodium valproate 400 mg IV q8 h; phenytoinum natricum 0.1, tid; phenobarbital sodium 0.2 IM, bid	Kaipulan 1.0, bid
6	Female	47	Complex partial seizure	Carbamazepine 0.2, bid; sodium valproate tablet, 500 mg, tid; sodium valproate injection, 400 mg IV, q12 h; phenobarbital sodium, 0.2 IM, bid; +sodium valproate tablet, 500 mg, tid; sodium valproate injection, 400 mg IV, q12 h	Kaipulan 1.0, bid
7	Female	48	Simple partial seizure secondary generalized tonic clonic seizure		Kaipulan 1.0, bid phenobarbital sodium 0.2 IM, bid
8	Male	48	Complex partial seizure+secondary generalized tonic clonic seizure	Sodium valproate 400 mg IV, q12 h, sodium valproate 500 mg, tid; Qulai 300 mg, bid	Kaipulan 0.5, bid
9	Female	49	Complex partial seizure+secondary generalized tonic clonic seizure	Carbamazepine 0.2, bid sodium valproate 400 mg, IV, q8 h; phenobarbital sodium 0.2 IM, bid	Kaipulan, 1.0, bid
10	Male	50	Simple partial seizure	VPA 500 mg, bid	
11	Female	45	Simple partial seizure	CBZ 0.2, bid VPA 400 mg IV, bid	LEV 0.5, bid phenobarbital sodium 0.2 IM, qd
12	Male	48	Complex partial seizure	CBZ 0.4, bid VPA 0.2, bid	LEV 0.5, bid phenobarbital sodium 0.2 IM, qd
13	Male	23	Simple partial seizure	LMT 100 mg, bid TP 75 mg, bid	LEV 0.5, bid VPA 400 mg IV, tid phenobarbital sodium 0.2 IM, qd

IV, intravenous; IM, intramuscular; VPA, valproic acid; CBZ, carba,azepine; LMT, lamotrigine.
